# 

**Published:** 1896-01

**Authors:** 


### INDEX TO VOLUME XXVI.

PAGE.

Adenoids in the Naso-Pharynx.............53
Atlanta Society of Medicine................72
An American Text-book of Obstetrics.....87
Antitoxin....................................94
American Text-book of Surgery..........138
Anesthesia, The History of..............140
Armstrong, W. S.........................144
Acetanalid as a Local Antiseptic........168
American Academy of Medicine...........196
American Medical Association............193
Abortion, Treatment of...................270
Anti-cholera Inoculation..................286
Aachen Treatment of Syphilis............292
Atlanta Meeting American Medical Association...........................301
Announcement Extraordinary.............468
Adeps Lanae, an Ideal Ointment Base....513
Anchoring the Kidney.....................600
Bladder, Tuberculosis of..................176
Burial, Premature.........................189
Bicycle, Two Dangers of..................345
Bromides, Some Unsuspected effects of...505
Beri-beri in the Fiji Islands..............460
Buffalo Lithia Water, Solvent Properties.624
Colles' Fracture...........................326
Case of Gunshot Wound of Stomach.....338
Chronic Gastritis of Long Standing......347
Case of Morphine and Opium Habit Cured...................................278
Cases of Aneroticism in Women..........282
Circumcision, Pertaining to..............215
Cooper, Wm. C............................243
Chloroform.................................121
Clinical Society of Maryland..............129
Chloroform in Labor......................59
Case of Phthisis Apparently Cured.......80
Consent in Surgical Operations...........82
Circumcision, Suggestion in the Operation of.....................................85
Dangers Which Threaten the Use of Cod-liver Oil..................................265
Diseases of the Rectum and Anus........589
Double Optic Neuritis.....................123
Doctors Should Not be so Modest........461
Epidemic Typhoid Fever..................406
Electricity in the Functional Neuroses...332
Enurisis and its Treatment................335
Editorial....................................
Epitome of the History of Pathology....535
Extra-Peritoneal Lipoma..................7
Glaucoma in Relation to General Practice.373
Gunshot Wound of Stomach...............338
Guaiacol as Antipyretic...................566

PAGE.

Hemorrhoids...............................67
History of Anesthesia....................140
Harris, Dr. N. O..........................192
How Shall We Feed the Baby.............450
Is the Diagnosis of Skin Disease Necessary.....................................438
Injuries of Eyeball, Some.................386
Infection and Intoxication Through Food 284
Infiltration Anesthesia, Improved Syringe for...............................127
Ligation of Left Subclavian Artery......1
Laparotomy at Eighty-one Years, A Successful..................................468
Labor, Chloroform in.....................59
Leprosy in the U. S.......................281
Lipoma, Extra-Peritoneal..................7
Malarial Origin of Rheumatism...........8
Mechanical Treatment of Heart Disease..289
Maryland Clinical Society.................179
Memorial of Dr. T. S. Powell.............157
Medical Association of Ga................220
Modern Treatment of Eclampsia..........394
New York Letter...........................67
Non-Operative Treatment of Urethral Stricture..................................559
Operation for Appendicitis................5
Optic Neuritis, Double....................123
Observation in European Surgery.........238
On the Preparation of Anatomical material.....................................393
Obstetrical Cases, Some,..................427
Placenta, The, How and When Delivered.432
Peroxide of Hydrogen.....................457
Puerperal Eclampsia......................495
Preparation of Anatomical material......393
Prescriptions..............................
Personal Equation in the Use of the Forceps......................................319
Penetrating Wounds of the Chest.........240
Passing of the Ovary......................242
Penis Amputation.........................175
Premature Burial..........................189
Resection of Gasserian Ganglion..........498
Richmond Academy of Medicine.....296, 556
Report of Cases Aneroticism in Women..282
Rare Syphilitic Infection, A..............64
Strangulated Hernia.......................441
Surgical Immunization....................481
Some of the Unsuspected Effects of Bromides.....................................505
Some Injuries of the Eyeball.............386
Spitting Nuisance, The....................355
Specialist—vs. General Practitioner......233

PAGE.

Surgical Treatment Retro-Deviation Uterus .......................173
Suggestion in Operation of Circumcision. 85
Syphilitic Infection, A Rare .............. 64
Summer Complaint....................273
Society Reports........................... 9
Southern Surgical and Gynecological Association...............................
Subscribers, To............................ 42
Special Notes ..............................
Shall Opticians Attempt to Fit Glasses..392
Sleeplessness..............................390
Tri-State Medical Society of Georgia, Alabama and Tennessee....................603
Treatment of Neuralgia, etc..............567
Treatment of Abortion....................270
Tuberculosis and Neoplasm of Bladder...176
Treatment of Skin Disfigurements by Electrolysis....................................211
Transplantation of Teeth..................240
Thyroid or Thyreoid.......................468
Urinary Analysis..........................105
Virulence of Seminal Fluid in Secondary Syphilis.................................. 343
When Shall the Gonorrhœics Marry......412
Why is the Negro Black...................565

### INDEX TO CONTRIBUTORS.

PAGE.

Blackford, C. M............................535
Bermingham, E. J.......................... 33
Brodnox, Ben H........................... 59
Corson, E. R..............................319
Corput, G. M..............................497
Evans, T. R...............................121
Gottheil, W. S............................ 64
Gaston, J. McF..................... { 157 480
Giuteras, Ramon...........................175
Hodges, J. A..............................505
Harrison, V. W............................441
Holladay, W. M............................427
Hutchins, M. B..................... { 211 438
Jones, L. H...............................105
Krim, J. M................................123
LeCato, G. W..............................111
Lofton, M................................ 168
Long, J. W................................270
Love, I. N................................215
Maddox, D. S..............................567
Murfree, J. B.............................432
Morgan, J. B..............................326
Patterson, R. L........................... 8
Poirier, P................................498
Pepper, Wm................................ 80
Phillips, S. L............................386
Reed, H...................................600
Rockwell, A. D............................332
Roberts, W. O............................. 5
Simms, G. K...............................589
Stewart, J. P............................. 65
Stirling, A. W............................373
Smith, A. A...............................495
Valentine, F. C...........................559
Wathen, W. H.............................. 7
Wyeth, J. A............................... 1
Winter, J. T....................... { 265 450
Wolff, B..................................513

### Editorial.

### TO SUBSCRIBERS.

The Southern Medical Record begins the twenty-sixth volume with this issue.

The present management contemplates making a number of important improvements in the journal during the year 1896, which will be of great value to the subscribers. Their efforts in this direction will be greatly facilitated if subscribers will pay promptly. It is necessary to have money to conduct the journal properly and to keep in the printer's good books.

A large number of subscriptions terminated with the December issue and statements have been mailed to those who are in arrears. The response has not been as prompt as could be desired, and we again call attention to these statements. We know that it is frequently inconvenient to respond at once to such calls, but we trust that our subscribers will not neglect us, and will lend us material aid by paying up at their earliest convenience. We wish it distinctly understood that Dr. Dan H. Howell is no longer connected with this journal in any capacity and that all subscriptions expiring after June, 1895, should be paid us. We constantly receive letters addressed to Dr. Howell, which frequently contain checks and post office orders made payable to him. We trust that our readers will hold in mind Dr. Howell's severance of all connection with the journal, and will thus save us much delay, confusion and annoyance.

Dr. Louis H. Jones has the business management of the Record and all matters relating to this department must be referred to him, when they will receive prompt and courteous attention.

We want to keep the good will of all the Record's old friends, and make many new ones.

---

We call our readers' attention to our premium offers on insert. We are enabled, by special arrangement with the publishers of these well-known magazines, to offer any one of them together with the Record for the price of the latter. If you do not wish the magazines, check off what instrument or book you desire, tear out the page and enclose it to us, together with remittance. These offers hold good to cash renewals as well as to new subscribers. Cash must accompany all orders. Make remittance by check, post office or express money order.

